# New Fair Multiparty Quantum Key Agreement Secure against Collusive Attacks

**DOI:** 10.1038/s41598-019-53524-4

**Published:** 2019-11-20

**Authors:** Zhiwei Sun, Rong Cheng, Chunhui Wu, Cai Zhang

**Affiliations:** 1 School of Artificial Intelligence, Shenzhen Polytechnic, Shenzhen, Guangdong 518055 China; 2 Center for Quantum Computing, Peng Cheng Laboratory, Shenzhen, 518055 China; 3 Department of Computer Science, Guangdong University of Finance, Guangzhou, 510521 P.R. China; 4 College of Mathematics and Informatics, South China Agricultural University, Guangzhou, 510642 China; 5 School of Electronic and Electrical Engineering, University of Leeds, Leeds, LS2 9JT UK

**Keywords:** Quantum physics, Quantum information

## Abstract

Fairness is an important standard needed to be considered in a secure quantum key agreement (QKA) protocol. However, it found that most of the quantum key agreement protocols in the travelling model are not fair, i.e., some of the dishonest participants can collaborate to predetermine the final key without being detected. Thus, how to construct a fair and secure key agreement protocol has obtained much attention. In this paper, a new fair multiparty QKA protocol that can resist the collusive attack is proposed. More specifically, we show that in a client-server scenario, it is possible for the clients to share a key and reveal nothing about what key has been agreed upon to the server. The server prepares quantum states for clients to encode messages to avoid the participants’ collusive attack. This construction improves on previous work, which requires either preparing multiple quantum resources by clients or two-way quantum communication. It is proven that the protocol does not reveal to any eavesdropper, including the server, what key has been agreed upon, and the dishonest participants can be prevented from collaborating to predetermine the final key.

## Introduction

It has been proven that Shor’s algorithm can factor a large number and calculate the discrete logarithms in polynomial time by using a quantum computer. With the development of research on quantum computers, small-scale quantum computers have already been created by large companies and organisations around the world. If large-scale quantum computers become available in the not-too-distant future, current public-key cryptosystems like RSA or elliptic curves will become insecure. To solve this problem, it is necessary to select new techniques that are not vulnerable to quantum computers and to design, analyse, and implement new cryptographic schemes based on these techniques.

Quantum cryptography is a study of carrying out cryptographic tasks using the properties of quantum mechanics. Quantum key distribution (QKD), as a famous instance of quantum cryptographic tasks, enjoys information-theoretical security to exchange the key. QKD can detect outside attacks aiming at learning about the secret key by measuring the quantum system. However, the uncertainty principle shows that measuring a quantum system will unavoidable disturb it, which provides a method of detecting the presence of eavesdropping. A quantum cryptographic protocol is secure if no information about the secret key is leaked; otherwise, it will be aborted. So far various subfields of quantum cryptography have emerged to offer different functions, such as quantum secure direct communication^1–9^, quantum private comparison^10–14^, quantum signature^15,16^, and quantum oblivious transfer^17^.

In the past years, quantum key agreement (QKA) protocols have received much attention in the quantum cryptography world. Compared with quantum key distribution where one sends a generated key to the other one, quantum key agreement allows multiple parties to collaborate to equally produce a shared key. The security of QKA requires that no partial corrupted parties can determine the shared key and no information about the shared key can be obtained by any eavesdropper. There were only two parties involved in quantum key agreement protocols when they were studied at the beginning^18–24^. Later, they are generalized to the scenario where multiple parties are considered^25–36^.

Unfortunately, Liu *et al*. showed that part of the parties in a multiparty QKA protocol can predetermine the final agreed key before the end of the protocol^37^. In other words, most of the existing QKA protocols cannot resist collusive attack. One reason that the collusive attack can succeed in multiparty QKA protocols is that the malicious participants can share the the initial prepared quantum states with each other. When two parties in the particular position, they can calculate the bitwise exclusive OR result of all the other’s secret key. With the result, they are able to predetermine the final agreed key. Thus, how to design a key agreement protocol which can be secure against collusive attack has obtained much attention^38–40^. On the other hand, a number of protocols have emerged where a user with limited quantum capabilities, delegates tasks to a server, who has the completely quantum power, which is known as delegated quantum computation^17^. Based on the idea of delegated quantum computation, we propose a multiparty QKA protocol in the client-server model.

## Preliminaries

Let us review the existing travelling-type multi-party quantum key agreement (MQKA) protocol and the collusive attacks. Suppose that *N* participants *P*_0_, …, *P*_*N*−1_ have secret bit-string keys *K*_0_, …, *K*_*N*−1_, respectively.

### Short review of the travelling-type MQKA protocol

We will review the travelling-type MQKA protocol here, which has been discussed in ref. ^37^.

In the preparation stage, *P*_*i*_ (*i* = 0, …, *N* − 1) generates many entangled states, each of which is then divided into two parts. One of them called “the home qubit sequence” (denoted as *R*_*i*_) will be kept, and the other one called “the travel qubit sequence” (denoted as *S*_*i*_) will be sent out. *P*_*i*_ then generates decoy particles that are later inserted into *S*_*i*_. The inserted *S*_*i*_ is denoted as ${S^{\prime} }_{i}$. *P*_0_, …, *P*_*N*−1_ stand in a circle such that *P*_*i*_’s neighborhoods are *P*_*i*−1*modN*_ and *P*_*i*+1*modN*_ (*P*_0_, …, *P*_*N*−1_). All the ${S^{\prime} }_{i}$ are sent to *P*_*i*+1*modN*_. When all the ${S^{\prime} }_{i}$ have been received by *P*_*i*+1*modN*_, they detect attacks and encode *K*_*i*+1*modN*_ into *S*_*i*_ (by removing decoy particles from *S*_*i*_)′). Afterward, decoy particle will be inserted into the encoded sequence and the new sequence will be sent to next participant. This process is similar to what *P*_*i*_ does in the previous step. Each participant repeats this process. After all participants finish the above process, *S*_*i*_ forms a complete circle. *S*_*i*_ is then measured by *P*_*i*_ who obtains *K*_0_ ⊕ *K*_1_ ⊕ … ⊕ *K*_*i*−1_ ⊕ *K*_*i*+1_ ⊕… ⊕ *K*_*N*−1_. Finally, all participants can get the shared key.

### Liu’s collusive attacks against CT-MQKA protocol

Liu’s collusive attacks^37^ consist of two stages. The first stage is the key-stealing stage and the the second stage is the key-flipping stage. In the first stage, the corrupted participants do their best to collaborate to computer the bitwise XOR outcome of the others’ secret keys by exploiting various quantum resources. In the second stage, they then change the encoded keys in accordance with the above outcome to determine a fake shared key.

It has been shown in ref. ^37^ that any two parties *P*_*i*_ and *P*_*j*_ (*i* > *j*) can control the shared key if the following conditions hold:

1 $$i-j=\frac{N}{2}\,\,{\rm{for}}\,{\rm{an}}\,{\rm{even}}\,{\rm{N}};$$

2 $$i-j=\frac{N-1}{2}\,{\rm{or}}\,\frac{N+1}{2}\,\,{\rm{for}}\,{\rm{an}}\,{\rm{odd}}\,{\rm{N}}.$$

Once Eq. (1) or Eq. (2) holds, the following attack can be launched by *P*_*i*_ and *P*_*j*_. For easy described the attack, suppose *N* is an even number.

1. **The key-stealing stage**:

- When the protocol starts, *P*_*i*_ and *P*_*j*_ share the knowledge of *R*_*i*_, *S*_*i*_, *K*_*i*_ and *R*_*j*_, *S*_*j*_, *K*_*j*_ and the expected fake key *K*′.
- In the (*i* − *j*)-th period when *P*_*j*_ starts the protocol, upon receiving *S*_*j*_, *P*_*i*_ is able to attain the bitwise XOR result of *K*_*j*+1_, *K*_*j*+2_, …, *K*_*i*−1_ according to the measurement outcomes of *R*_*j*_ and *S*_*j*_. Analogously, *P*_*j*_ could obtain the XOR result of *K*_*i*+1_, *K*_*i*+2_, …, *K*_*j*−1_ in the (*N* − *i* + *j*)-th period when *P*_*i*_ starts the protocol.
- *P*_*i*_ and *P*_*j*_ exchange the above bitwise XOR results. Then, they can compute the legal shared key *K* in the *i* − *j* period in advance.

2. **The key-flipping stage**:

- Suppose *K*′ is the fake key that collusive participants want to share. In the *i* − *j* period, *P*_*i*_ and *P*_*j*_ encode ${K^{\prime} }_{i}={K}_{i}\oplus K^{\prime} \oplus K$ instead of *K*_*i*_, and ${K^{\prime} }_{j}={K}_{j}\oplus K^{\prime} \oplus K$ instead of *K*_*j*_ respectively. One can verify that any participant will obtain the fake final shared key *K*′.

## Results

### The proposed fair multiparty QKA protocol

In the above attack, any two malicious parties *P*_*i*_ and *P*_*j*_ in particular positions can exchange the information about their initial prepared quantum states. Then they can collaborate with other to compute the the final shared key *K* before the last period. They can finally predetermine the fake key based on these information. Most MQKA protocols are therefore insecure against collusive attacks. To achieve the fairness property, two conditions should be removed. The first one is that the information about the initial prepared states cannot be shared among collusive parties. Without these information, any two malicious parties can obtain nothing about other parties’ keys. Thus, they cannot compute the final shared key *K* in advance. There is no way for them to generate a fake final shared key. In order to launch Liu’s attack^37^, all quantum states generated by the honest parties should pass the malicious parties at least once. The situations in Sun’s protocols^33,38^, are a little different. The travelling model is divided into parts. Since the malicious parties are limited to only part of information about the other parties’ keys before the last period, which makes them fail to computer the bitwise XOR outcomes of all the other’s secret keys any more. In such way, Sun’s protocols are secure against *t*-party collusive attacks. Here, *t* < *N*.

The first method will be employed to devise a fair MQKA protocol in this work. To make the collusive parties share nothing about the initial prepared states among them, these parties are restricted to generating initial states. The stage of initial states is delegated to a server. The server plays a role of generating the initial states, forwarding them to parties and announcing the generated initial states in the last period via authenticated classical channels. We assume that the server is semi-honest. In other words, the server will honestly follow the protocol and cannot collude with any other party but she may try to learn about extra information about the parties’ secret keys, other than what the process of the protocol naturally implies. The parties are then only required to make measurements and do unitary operations. We also assume that the classical channels in our protocol are authenticated and the quantum channels are lossless and noiseless.

Suppose participants *P*_1_, …, *P*_*N*−1_ have secret *m*-bit keys *K*_0_, …, *K*_*N*−1_, respectively, they intend to generated a shared key *K* such that *K* = *K*_0_ ⊕ … ⊕ *K*_*i*_ ⊕ … ⊕ *K*_*N*−1_. The participants stand in a circle in the following way: *P*_*i*_ has *P*_*i*−1_ and *P*_*i*+1_ as his left and right neighbors, respectively, where *P*_*i*±*N*_ = *P*_*i*_ for 0 < = *i* < *N*.

Generally, our protocol will reveal nothing about the shared key to any eavesdropper, including the server. And it is also secure against the collusive attacks.

The detailed steps of our protocol can be described in the following:

1. **Preparation stage:** The server prepares *N* sequences $\{{S}_{0},\cdots ,{S}_{N-1}\}$, which are called the message sequences. Sequence ${S}_{i},i=0,\cdots ,N-1$ consists of *m* ordered single photons. Each single photon is randomly selected from $\{|0\rangle ,|1\rangle ,|\,+\,\rangle ,|\,-\,\rangle \}$. To check for eavesdropping, the server prepares another *N* sequences $\{{C}_{0},\cdots ,{C}_{N-1}\}$ which is called the decoy sequence, and the decoy sequence ${C}_{i},(i=0,\cdots ,N-1$) consists of *m* ordered single photons. The single photon is randomly in one of the states $\{|\,+\,\rangle ,|\,-\,\rangle ,|\,+\,y\rangle ,|\,-\,y\rangle \}$. Here, $|\,\pm \,\rangle =\frac{1}{\sqrt{2}}(|0\rangle \pm |1\rangle ),|\,\pm \,y\rangle =\frac{1}{\sqrt{2}}(|0\rangle \pm i|1\rangle )$, which are called decoy states. For all $i=0,\cdots ,N-1$, the server randomly inserts *C*_*i*_ into *S*_*i*_ to get a new sequence ${S^{\prime} }_{i}$ which is called the travelling sequence, and sends ${S^{\prime} }_{i}$ to *P*_*i*_.
2. **Detection stage:** After confirming that all the *N* parties, ${P}_{0},\cdots ,{P}_{N-1}$, have received the message sequences sent from the server, the server publishes the positions and corresponding bases of the decoy sequence in the travelling sequence. Based on these information, for $i=0,\cdots ,N-1$, *P*_*i*_ can measure *C*_*i*_ in the correct bases. Then, he/she stores the measurement results and randomly publishes half of the measurement outcomes. Correspondingly, the server publishes the information of the initial states of the other half of *C*_*i*_. By comparing the measurement results of the decoy sequence with their corresponding initial states, the server and *P*_*i*_ can calculate the error rate. If the error rate is lower than the predetermined threshold value, the protocol will be proceeded; otherwise, the protocol will be aborted and restarted from Step 1.
3. After the detection stage, *P*_*i*_ obtains the secure travelling sequence *S*_*i*_. Here, $i=0,\cdots ,N-1$. Each party ${P}_{i},i=0,\cdots ,N-1$, performs the following steps:

1. **Encoding stage:** *P*_*i*_ encodes *K*_*i*_ onto *S*_*i*_ by the following encoding rule: when the classical bit of the *k*_*i*_ is 1, the unitary operation $U=|0\rangle \langle 1|-|1\rangle \langle 0|$ is performed onto *S*_*i*_. Otherwise, the identity unitary operation $I=|0\rangle \langle 0|+|1\rangle \langle 1|$ is made. The role of unitary operator *U* is to flip the quantum states, in other words, $U|0\rangle =-\,|1\rangle ,U|1\rangle =|0\rangle ,U|+\rangle =|\,-\,\rangle ,U|-\rangle =-\,|\,+\,\rangle$. Then *P*_*i*_ rearranges the *m* decoy states generated by server in Step 1, and randomly inserts them into the encoded sequence to get a new one which is denoted as ${S}_{i}^{i+1}$. After the above encoding stage, *P*_*i*_ forwards ${S}_{i}^{i+1}$ to *P*_*i*+1_.
2. **Eavesdropping check stage:** The eavesdropping check stage is similar to the server and *P*_*i*_ did in Step 2. In other words, when *P*_*i*+1_ has received the sequence ${S}_{i}^{i+1}$ from *P*_*i*_, *P*_*i*_ tells *P*_*i*+1_ the decoy states’ positions and the corresponding bases in the sequence ${S}_{i}^{i+1}$. According to these information, *P*_*i*+1_ measures the decoy sequence in the corresponding correct bases, stores them and randomly announces half of the measurement result. Then, *P*_*i*_ publishes the initial states of the other half decoy sequence. According to the announced information, i.e., the measurement results of the decoy sequence and the initial decoy sequence, they can calculate the error rate. If the error rate is lower than the predetermined threshold value, the protocol will be proceeded; otherwise, the protocol will be aborted and restarted from Step 1.
3. **Encoding stage:** After the detection phase, *P*_*i*+1_ obtains the message sequence *S*_*i*_. He then encodes *K*_*i*+1_ onto *S*_*i*_ by the encoding rule in Step (1). Then *P*_*i*+1_ rearranges the *m* decoy states, and randomly inserts the decoy states into the encoded sequence to get a new one which is denoted as ${S}_{i}^{i+2}$. After the above encoding stage, *P*_*i*+1_ forwards ${S}_{i}^{i+2}$ to *P*_*i*+2_.
4. Then, the *N* − 1 participants, ${P}_{i+2},{P}_{i+3},\cdots ,{P}_{i-2}$ repeatedly execute the eavesdropping check stage and encoding state in the same way as in Steps (2) and (3).
5. When *P*_*i*−1_ receives ${S}_{i}^{i-2}$ from *P*_*i*−2_, *P*_*i*−1_ and *P*_*i*−2_ check for eavesdropping with the decoy states method. If the transmission is secure, *P*_*i*−1_ discards the decoy states and obtains the secure message sequence *S*_*i*_, and he announces this fact to server.

4. Once all the *P*_*i*−1_ obtains the secure message sequence *S*_*i*_, the server announces the positions and corresponding bases of the *S*_*i*_ to *P*_*i*−1_. For each participant *P*_*i*−1_, he then measures each of the message sequence *S*_*i*_ in the corresponding bases to obtain an *m*-bit string ${K^{\prime} }_{i}={K}_{i}\oplus {K}_{i+1}\oplus \cdots \oplus {K}_{i-2}$. Then *P*_*i*−1_ can deduce the final shared key ${K}_{i}^{\text{'}}\oplus {K}_{i-1}={K}_{0}\oplus \cdots \oplus {K}_{i}\oplus \cdots \oplus {K}_{N-1}=K$. Here, $i=0,\cdots ,N-1$.

Note that the above protocol is considered in the semi-honest model, if there are malicious parties, the shared key *K* may be not identical. In order to prevent them from fooling the honest one, the *N* participants ${P}_{0},\cdots ,{P}_{N-1}$ can randomly select parts of the *K* to detect eavesdropping. If there is no malicious party, the rest of the *K* will be the final shared key. The following section will discuss the security analysis of the presented protocol.

### Security analysis of the proposed protocol

First, we prove that the proposed protocol is secure against external eavesdropping. Then, we show that it is immune to attacks from internal eavesdropping.

#### Security against external eavesdropping

To detect outside eavesdropping, the decoy-state method is used in the presented protocol. The decoy-state method uses several non-orthogonal single states, $|\,+\,\rangle ,|\,-\,\rangle ,|\,+\,y\rangle ,|\,-\,y\rangle$, which are randomly inserted in the message sequence. Because of quantum indistinguishability, Eve cannot distinguish between the message sequence and the decoy states. The Eve may apply the same operation on all the quantum states. Usually, the operation Eve makes is denoted as *U*_*E*_ which causes the message sequence to interact coherently with an auxiliary quantum system $|E\rangle$, which can be denoted as follows:

3 $${U}_{E}|0\rangle |E\rangle =a|0\rangle |{E}_{00}\rangle +b|1\rangle |{E}_{01}\rangle ,$$

4 $${U}_{E}|1\rangle |E\rangle =c|0\rangle |{E}_{10}\rangle +d|1\rangle |{E}_{11}\rangle ,$$

where $|a{|}^{2}+|b{|}^{2}=1$ and $|c{|}^{2}+|d{|}^{2}=1$. In the following part, we will prove that any malicious behavior by Eve will inevitably modify the photon statistic and expose her.

Since the decoy states involved in our protocol are $|\,+\,\rangle$, $|\,-\,\rangle$, $|\,+\,y\rangle$ and $|\,-\,y\rangle$, if Eve introduces no error in the eavesdropping check by participants, the general operation *U*_*E*_ must satisfy the following conditions:

5 $$\begin{array}{rcl}{U}_{E}|+\rangle |E\rangle  & = & \frac{1}{\sqrt{2}}(a|0\rangle |{E}_{00}\rangle +b|1\rangle |{E}_{01}\rangle +c|0\rangle |{E}_{10}\rangle +d|1\rangle |{E}_{11}\rangle )\\  & = & \frac{1}{2}(|\,+\,\rangle (a|{E}_{00}\rangle +b|{E}_{01}\rangle +c|{E}_{10}\rangle +d|{E}_{11}\rangle ))\\  & + & \frac{1}{2}(|\,-\,\rangle (a|{E}_{00}\rangle -b|{E}_{01}\rangle +c|{E}_{10}\rangle -d|{E}_{11}\rangle ))\\  & = & \frac{1}{2}(|\,+\,\rangle (a|{E}_{00}\rangle +b|{E}_{01}\rangle +c|{E}_{10}\rangle +d|{E}_{11}\rangle )).\end{array}$$

6 $$\begin{array}{c}\begin{array}{rcl}{U}_{E}|-\rangle |E\rangle  & = & \frac{1}{\sqrt{2}}(a|0\rangle |{E}_{00}\rangle +b|1\rangle |{E}_{01}\rangle -c|0\rangle |{E}_{10}\rangle -d|1\rangle |{E}_{11}\rangle )\\  & = & \frac{1}{2}(|\,+\,\rangle (a|{E}_{00}\rangle +b|{E}_{01}\rangle -c|{E}_{10}\rangle -d|{E}_{11}\rangle ))\\  & + & \frac{1}{2}(|\,-\,\rangle (a|{E}_{00}\rangle -b|{E}_{01}\rangle -c|{E}_{10}\rangle +d|{E}_{11}\rangle ))\\  & = & \frac{1}{2}(|\,-\,\rangle (a|{E}_{00}\rangle -b|{E}_{01}\rangle -c|{E}_{10}\rangle +d|{E}_{11}\rangle )).\end{array}\end{array}$$

7 $$\begin{array}{c}\begin{array}{rcl}{U}_{E}|\,+\,y\rangle |E\rangle  & = & \frac{1}{\sqrt{2}}(a|0\rangle |{E}_{00}\rangle +b|1\rangle |{E}_{01}\rangle +ic|0\rangle |{E}_{10}\rangle +id|1\rangle |{E}_{11}\rangle )\\  & = & \frac{1}{2}(|\,+\,y\rangle (a|{E}_{00}\rangle -ib|{E}_{01}\rangle +ic|{E}_{10}\rangle +d|{E}_{11}\rangle ))\\  & + & \frac{1}{2}(|\,-\,y\rangle (a|{E}_{00}\rangle +ib|{E}_{01}\rangle +ic|{E}_{10}\rangle -d|{E}_{11}\rangle ))\\  & = & \frac{1}{2}(|\,+\,y\rangle (a|{E}_{00}\rangle -ib|{E}_{01}\rangle +ic|{E}_{10}\rangle +d|{E}_{11}\rangle )).\end{array}\end{array}$$

8 $$\begin{array}{rcl}{U}_{E}|\,-\,y\rangle |E\rangle  & = & \frac{1}{\sqrt{2}}(a|0\rangle |{E}_{00}\rangle +b|1\rangle |{E}_{01}\rangle -ic|0\rangle |{E}_{10}\rangle -id|1\rangle |{E}_{11}\rangle )\\  & = & \frac{1}{2}(|\,+\,y\rangle (a|{E}_{00}\rangle -ib|{E}_{01}\rangle -ic|{E}_{10}\rangle -d|{E}_{11}\rangle ))\\  & + & \frac{1}{2}(|\,-\,y\rangle (a|{E}_{00}\rangle +ib|{E}_{01}\rangle -ic|{E}_{10}\rangle +d|{E}_{11}\rangle ))\\  & = & \frac{1}{2}(|\,-\,y\rangle (a|{E}_{00}\rangle +ib|{E}_{01}\rangle -ic|{E}_{10}\rangle +d|{E}_{11}\rangle )).\end{array}$$

From the above Eqs. (5–8), we can get

9 $$a|{E}_{00}\rangle -b|{E}_{01}\rangle +c|{E}_{10}\rangle -d|{E}_{11}\rangle =0,$$

10 $$a|{E}_{00}\rangle +b|{E}_{01}\rangle -c|{E}_{10}\rangle -d|{E}_{11}\rangle =0,$$

11 $$a|{E}_{00}\rangle +ib|{E}_{01}\rangle +ic|{E}_{10}\rangle -d|{E}_{11}\rangle =0,$$

12 $$a|{E}_{00}\rangle -ib|{E}_{01}\rangle -ic|{E}_{10}\rangle -d|{E}_{11}\rangle =0.$$

Here 0 denotes a column zero vector. Further, we can get *a* = *d* = 1, *b* = *c* = 0 and $|{E}_{00}\rangle =|{E}_{11}\rangle$. Therefore,

13 $${U}_{E}|0\rangle |E\rangle =|0\rangle |{E}_{00}\rangle ,$$

14 $${U}_{E}|1\rangle |E\rangle =|1\rangle |{E}_{00}\rangle ,$$

15 $${U}_{E}|\,+\,\rangle |E\rangle =|\,+\,\rangle |{E}_{00}\rangle ,$$

16 $${U}_{E}|\,-\,\rangle |E\rangle =|\,-\,\rangle |{E}_{00}\rangle ,$$

i.e., Eve introduces no error in the eavesdropping only when her ancillary state and the target photon $\{|0\rangle ,|1\rangle ,|\,+\,\rangle ,|\,-\,\rangle \}$ are product states. So outside eavesdroppers cannot obtain the shared key without being detected. In addition, each transmission of the qubit sequences is not a closed ring, i.e., the transmission is not a two-way quantum channel any more. Thus, the Trojan horse and invisible photon attacks can be naturally resisted.

#### Security against internal eavesdropping

As known to all, the dishonest parties in a protocol have more power than those from external eavesdroppers to attack the protocol. The dishonest parties can lie in the eavesdropping check stage or substitute the message sequence with their desired message sequence in order to predetermine the final shared key. Thus, all the proposed QKA protocols need to be secure against internal dishonest parites’ attack.

Liu’s collusive attack can be divided into two stages^37^: the key stealing stage and the key flipping stage. The key stealing stage or the key flipping stage must be destroyed in order to design a secure QKA protocol. In this paper, the proposed protocol which is secure in the stealing stage is analyzed as follows:

We first consider the worst case that there are *N* − 1 dishonest parties and only one honest party, ${P}_{t},t\in \{0,\cdots ,N-1\}$. In order to predetermine the final shared key, the *N* − 1 dishonest parties need to obtain ${P^{\prime} }_{t}s$ private key *K*_*t*_ before ${P^{\prime} }_{t+1}s$ quantum sequence *S*_*t*+1_ is sent to *P*_*t*_. If the dishonest parties have already obtained *K*_*t*_, they can launch the following attack: When TP sends the message sequence *S*_*t*+1_ to *P*_*t*+1_. Then, the *N* − 1 dishonest participant ${P}_{t+1},\cdots ,{P}_{t-1}$ just forward the message sequence *S*_*t*+1_ to the next one using the decoy method. If *P*_*t*−1_ receives *S*_*t*+1_ from *P*_*t*−2_, after the eavesdropping check stage, *P*_*t*−1_ encodes *K*_*t*_ ⊕ *K*′ onto *S*_*t*+1_, and sends the new sequence to *P*_*t*_ in the secure way. When the server announces the positions and corresponding bases of the *S*_*t*+1_ to *P*_*t*_ in step 4, it is easy to verify that the final key *P*_*t*_ obtained is the fake key *K*_*t*_ ⊕ *K*_*t*_ ⊕ *K*′ = *K*′.

Fortunately, we will show that it is impossible to obtain ${P^{\prime} }_{t}s$ private key *K*_*t*_ before ${P^{\prime} }_{t+1}s$ quantum sequence *S*_*t*+1_ is sent to *P*_*t*_ in our protocol. Since the initial quantum states are prepared by server and the server honestly executes the protocol and does not cooperate with any participant. He will not leak any information about the initial prepared quantum states to any participant before the step 4. In order to obtain *K*_*t*_, the only way for the dishonest parties is to measure the message sequence just like the external Eve does. However, security against external eavesdropping has been proven in the above subsection. Thus, this kind of internal attack can be prevented.

Secondly, some dishonest parties may just intend to fool some parties, making the legitimate parties accept the fake key *K*′ as the final shared key *K*. For example, when TP sends the message sequence *S*_*t*+1_ to *P*_*t*+1_, the dishonest *P*_*t*+1_ can encode *K*_*t*+1_ ⊕ *K*_*f*_ in the encoding stage in order to fool the honest party *P*_*t*_. Here, $t\in \{0,\cdots ,N-1\}$, the key *K*_*f*_ is used to fool *P*_*t*_. When the server announces the positions and corresponding bases of the *S*_*t*+1_ to *P*_*t*_ in step 4, it is easy to verify that the final shared key of *P*_*t*_ is the fake key *K*_*f*_ ⊕ *K*. In order to detect the malicious behavior of the dishonest parties, the *N* parties can randomly choose part of the final shared key *K* to detect the error when they have already obtained *K*. If the error rate is higher than predetermined value, the protocol is abort. Otherwise, the rest bit of *K* is used for the final key.The details can be found in ref.^41^.

Thirdly, the server may also try to learn extra information about participants’ secret key from the protocol execution. Notice that the presented protocol is a one-way quantum channel, the server prepares the initial quantum states and sends them to the participant, but these quantum states will not be sent back to server. Thus, if the server tries to learn extra information about participants’ secret key, he/she may need to measure the quantum channel, just like the external attackers do. Because of the decoy states method, this attack can be detected in the presented protocol. Thus, the server cannot get any information about the parties’ secret key. If the server uses Trojan horse or invisible photon attacks, the method in ref.^42^ can be used to resist these attacks.

### Efficiency

In this section, we compare the qubit efficiency of different MQKA protocols. The qubit efficiency is defined as $\eta =\frac{c}{q+b}$ ^41^. Here, *c* represents the length of the final shared key, *q* denotes the number of the qubits required for encoding and eavesdropping process and *b* refers to the number of bits needed for decoding process.

**Figure 1:**
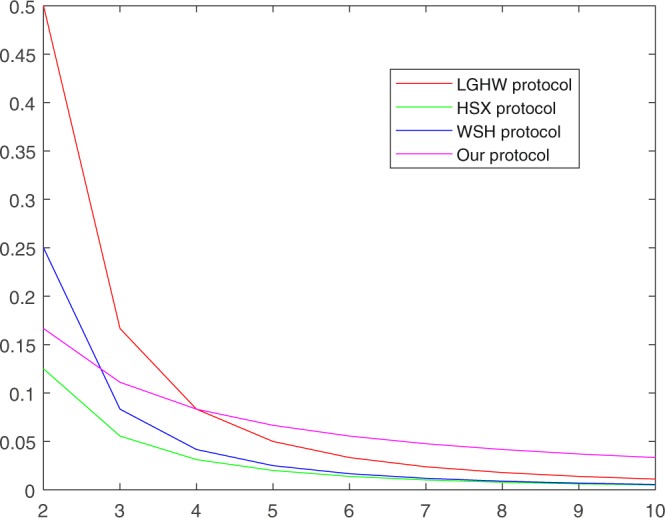
Efficiency comparison of the different protocols, where *κ*  = 1 and *N*  = 2, 3, …, 10.

In our *N*-party QKA protocol, in order to share a *m*-bit secret key, *m* single photons are used, and *m* decoy qubits are required in every transmission and *N* rounds of transmission are involved. Totally, *N*(*m* + *m*) qubits should be required. The server announces *mN* bits to the parties to decode the shared key. The qubit efficiency is therefore $\eta =\frac{m}{(m+m)N+mN}=\frac{1}{3N}$. However, in order to be secure against collusive attacks, the proposed protocol needs the server’s help. Meanwhile, the initial qubits preparation is delegated to the server, while participants just make measurement and do unitary operations on them, which makes our protocol more practical. Table 1 shows the efficiency comparison of our protocol and several existing secure MQKA protocols. As we can see in Fig. 1, if there are more than four parties involved in MQKA protocols, our protocol efficiency becomes much better than that of other protocols.

**Table 1:** Efficiency comparison. For easier comparison, let the key length is *m*, the number of participants is *N*, the detection rate *κ*  = 1, the dishonest participants *t*  =  *N* − 1.

QKA protocol	*η*	participants prepare message states	TP	Collusive Attacks
LGHW protocol	$\frac{1}{(N-1)N}$	*mN* (*N* − 1)	No	Secure
HSX protocol	$\frac{1}{2{N}^{2}}$	*mN*	No	Secure
WSH protocol	$\frac{1}{2(N-1)N}$	*mN* (*N* − 1)	No	Secure
Ours	$\frac{1}{3N}$	0	Yes	Secure

## Conclusion

In conclusion, we proposed a multiparty quantum key agreement protocol which can resist Liu’s collusion attack which is presented in the ref.^37^. To prevent the Liu’s attack, the participants are restricted to preparing the initial quantum states in the proposed protocol. The stage of initial quantum states preparation is delegated to a server. It is proven that the protocol does not reveal the final shared key to any eavesdropper, including the server. And the participants involved in the protocol no longer need to prepare quantum states for message encoding, which makes the protocol more practical. And the main contribution of the paper is that we proposed a new model for quantum key agreement in client-server model, which protects the honest participants’ fairness.
